# The Role of Adiponectin during Pregnancy and Gestational Diabetes

**DOI:** 10.3390/life13020301

**Published:** 2023-01-21

**Authors:** Brittany L. Moyce Gruber, Vernon W. Dolinsky

**Affiliations:** 1 Diabetes Research Envisioned and Accomplished in Manitoba (DREAM), Research Theme of the Children’s Hospital Research Institute of Manitoba, Winnipeg, MB R3E 3P4, Canada; 2 Department of Pharmacology and Therapeutics, University of Manitoba, Winnipeg, MB R3E 0T6, Canada

**Keywords:** adiponectin, pregnancy, gestational diabetes

## Abstract

Pregnancy involves a range of metabolic adaptations to supply adequate energy for fetal growth and development. Gestational diabetes (GDM) is defined as hyperglycemia with first onset during pregnancy. GDM is a recognized risk factor for both pregnancy complications and long-term maternal and offspring risk of cardiometabolic disease development. While pregnancy changes maternal metabolism, GDM can be viewed as a maladaptation by maternal systems to pregnancy, which may include mechanisms such as insufficient insulin secretion, dysregulated hepatic glucose output, mitochondrial dysfunction and lipotoxicity. Adiponectin is an adipose-tissue-derived adipokine that circulates in the body and regulates a diverse range of physiologic mechanisms including energy metabolism and insulin sensitivity. In pregnant women, circulating adiponectin levels decrease correspondingly with insulin sensitivity, and adiponectin levels are low in GDM. In this review, we summarize the current state of knowledge about metabolic adaptations to pregnancy and the role of adiponectin in these processes, with a focus on GDM. Recent studies from rodent model systems have clarified that adiponectin deficiency during pregnancy contributes to GDM development. The upregulation of adiponectin alleviates hyperglycemia in pregnant mice, although much remains to be understood for adiponectin to be utilized clinically for GDM.

## 1. Metabolic Adaptations during Pregnancy

In this review, we summarize the current state of knowledge about the role of adiponectin and its intracellular signalling pathways in metabolic adaptations to pregnancy. Because clinical evidence has shown a correlative association between low circulating adiponectin levels and gestational diabetes mellitus (GDM), we chose to focus this review on recent studies using pregnant female adiponectin knockout mice, showing several metabolic features of GDM, suggesting that adiponectin deficiency may have a role in GDM development. We also review additional studies in mice examining how increasing adiponectin levels during pregnancy impact insulin sensitivity and could be a treatment for GDM.

**Figure 1:**
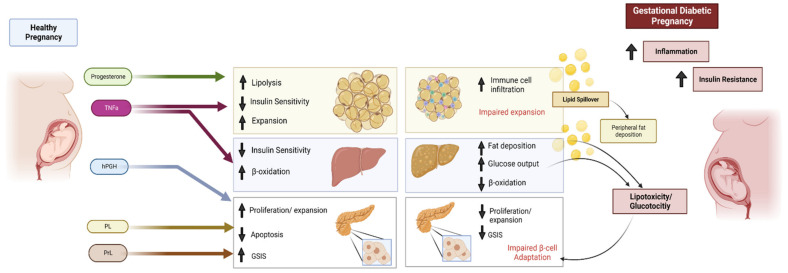
Metabolic adaptations of late gestation and the impact of gestational diabetes. ↑ increased, ↓ decreased. Created with BioRender.com.

In mammals, several adaptations are required to sustain pregnancy. For instance, the maternal system exhibits increased blood volume and cardiac output accompanied by a corresponding increase in renal activity [1]. In addition, increased respiratory capacity and neurological changes, specifically increased neural plasticity and increased neurogenesis also occur during pregnancy and lactation [1,2]. Furthermore, metabolic adaptations are a necessary response to the changing metabolic demands of the mother and fetus (Figure 1). These can be broadly divided into two phases. In the first phase, which corresponds to the first two trimesters in a human pregnancy, there is an overall anabolic period. During this stage, the maternal system builds up energy stores, increasing lipid storage in tissues to prepare for increased breakdown and use later in pregnancy [3,4]. To achieve this, maternal energy consumption increases, as does *de novo* hepatic lipogenesis. In the later stages of pregnancy, lipid deposits are preferentially broken down for fatty acid oxidation, which spares glucose for fetal growth [3,4,5,6].

Many hormonal, metabolic and immunological changes, including insulin resistance, are set in motion to adequately adapt to maternal and fetal needs during gestation (reviewed in [7,8,9]). Hormones such as human placental growth hormone (hPGH) increase progressively throughout pregnancy [10] and reduce signalling through the insulin receptor substrate–1(IRS-1), as well as glucose transporter (GLUT)-4 mediated glucose uptake. Later in gestation, maternal metabolism shifts to a catabolic phase and lipids are transiently increased, and mild hyperinsulinemia occurs. Despite these changes, in most pregnancies, normoglycemia is maintained and, in some cases, decreases, which could be due to the dilution effect as blood volume increases in the maternal system [11]. Additionally, increased insulin production and secretion by the maternal pancreas contribute to the maintenance of maternal glycemia [12]. In pregnancy, peripheral tissues become more insulin-resistant [3,13], and hepatic glucose output is not suppressed by insulin. In order to achieve glucose homeostasis in a healthy pregnancy, circulating insulin is increased to overcome insulin resistance [4]. Postnatally, these hormonal and metabolic changes return to baseline [14].

Changes to circulating hormones and cytokines in pregnancy impact insulin tolerance of peripheral tissues and compensatory adaptations of pancreatic β-cells that include increased proliferation, reduced apoptosis and enhanced glucose-stimulated insulin secretion (GSIS) [15]. In the third trimester, progesterone increases and may have a role in increasing adipose tissue lipolysis and decreasing insulin sensitivity and glucose uptake in peripheral tissues, including adipose tissue [16,17]. Increased lipolysis from adipose tissue may lead to increased fatty acid uptake by the liver and reduced hepatic insulin sensitivity. Human placental growth hormone (hPGH) increases significantly throughout gestation and has a role in stimulating pregnancy-induced β-cell adaptations [18,19]. Prolactin (PrL) and placental lactogen (PL) have been implicated in pregnancy-associated β-cell adaptations [20], as well as in peripheral insulin resistance and increased lipolysis [21]. Tumour necrosis factor (TNF)-α increases in the circulation towards the end of pregnancy and has been shown to mediate the insulin resistance that occurs in the third trimester of pregnancy [22,23]. In GDM, more severe insulin resistance occurs, as well as increased inflammation and lipotoxicity, which lead to impairments in the necessary adaptations by pancreatic β cells [15]. Additionally, impaired insulin sensitivity in the liver leads to increased hepatic glucose output and reduced β oxidation. Lipid spillover from adipose tissue because of impaired expandability can lead to hepatic fat deposition and lipotoxicity in both the pancreas and the liver [24,25].

### 1.1. The Endocrine Pancreas and Its Adaptation to Pregnancy

Islet architecture varies between species, but mice and rats have relatively well-defined pancreatic islet structures, with abundant β-cells (in mice, making up 60–80% of cells in the islet) and scarcer polypeptide (PP) cells, δ-cells and α-cells [26,27,28]. Studies in human and animal models have provided evidence for plasticity of the pancreatic islet during pregnancy [12,27,29], which allows for increases in mass and hormone secretory capacity to compensate for the metabolic demands of pregnancy. This research has largely focused on β-cells and, to a lesser extent, α-cells due to their role in the management of blood glucose levels.

The pancreatic islet responds to the demands of pregnancy involving a range of adaptive mechanisms, including β-cell hyperplasia and hyper-functionality, that are driven by transcription factors and cell cycle regulators [15,30,31,32]. Pregnancy-induced β-cell expansion is achieved by proliferation, hypertrophic expansion and possibly neogenesis from progenitor cells; these mechanisms are accompanied by a temporary reduction in apoptosis [12,30,33] that has also been observed in islets during human pregnancy [12]. These changes may be mediated by crosstalk between increased placental signalling, the maternal pancreas and peripheral tissues, which have been illustrated by the use of animal models [34,35,36]. While some adaptive mechanisms are conserved between species, there are variations in the primary adaptive responses between rodent and human islets in pregnancy. For instance, in rodent models of pregnancy, neogenesis may only contribute a small amount to the expansion of β-cell mass, with proliferation playing a more significant role [37]. In human pregnancy, neogenesis may exceed proliferation as an adaptive mechanism [33,38]. However, human islets isolated during pregnancy are scarce, and samples were heterogenous in origin (from pregnancies of varying gestational length, maternal ages, ethnicities and causes of death), complicating the interpretation [12]. Despite the heterogeneity of available samples, human islets isolated during pregnancy show increases in β-cell area relative to non-pregnant controls [12,29].

In mice, pregnancy also increases alpha-cell mass, pancreatic glucagon-like peptide (GLP-1), and pancreatic and circulating glucagon levels [39] in association with a transient increase in serum glucagon, which was previously reported in human pregnancy [40]. Additionally, pregnant mice lacking α-cells were reported to exhibit impaired glucose tolerance, which was rescued by administration of glucagon-like peptide (GLP)-1 receptor agonists [39], illustrating a role of α-cells in insulin secretion and islet adaptation to pregnancy. Hormones secreted by cells adjacent to β-cells provide an additional layer of control over insulin secretion [41], which is only beginning to be explored in pregnancy.

### 1.2. White Adipose Tissue in Pregnancy

Maternal white adipose tissue (WAT) undergoes expansion during early pregnancy, providing energy stores of fat as fuel for later stages of pregnancy. High levels of insulin increase de novo lipogenesis, suppress adipose tissue lipolysis [7,42] and lower circulating free fatty acids [43]. Because a significant amount of glucose is utilized during the early stages of pregnancy, insulin increases glucose transport from serum and increases glycolysis. There is also evidence that crosstalk with the placenta during pregnancy may promote adipose tissue expansion [44]. Previously, it was found that during pregnancy, increased plasma protein A (PAPP-A) stimulates proteolysis of insulin-like growth factor (IGF)-binding protein-5, which releases IGF-1 and promotes adipose tissue expansion and proliferation to enable energy storage and prevent excess ectopic lipid deposition elsewhere in the body [45]. A follow-up study in mice showed that a lack of PAPP-A impaired adipose tissue expansion, pregnancy-induced insulin resistance and ectopic fat deposition in the form of hepatic steatosis—all factors that are associated with a GDM phenotype [46]. Expansion of adipose tissue can be achieved through hypertrophic adipocytes (to accommodate larger lipid droplets) or hyperplasia, which is the production of new adipocytes from precursors [45]. However, all forms of adipose tissue expansion are not equal; hyperplasia is linked to better glucose control, whereas hypertrophy is associated with more severe insulin resistance and is more prone to inflammation [47].

As pregnancy progresses, maternal glucose utilization is reduced, and maternal metabolism shifts into a catabolic phase, during which adipose tissue energy stores are broken down [43,48,49]. Steady increases in circulating free fatty acid (FFA) and glycerol, as well as decreases in adipocyte size and WAT mass, occur towards the end of gestation. During this phase of pregnancy, maternal metabolism relies more heavily on fatty acid oxidation for energetic needs, so the increased availability of lipids is an important physiological adaptation to late gestation. Indeed, hormonal changes associated with pregnancy, including circulating human placental lactogen (HPL) and increased TNF-α increase insulin resistance, thereby reducing the inhibitory effects of insulin on lipolysis [50].

### 1.3. Adaptations of Liver Metabolism during Pregnancy

The liver plays a significant role in maintaining maternal glucose homeostasis throughout gestation. Alterations in hepatic insulin signalling control these processes. In early pregnancy, when the metabolic demands of fetal growth are low, hepatic insulin sensitivity remains high, hepatic gluconeogenesis is inhibited by insulin and glucose is utilized for maternal energy [7,51]. In late pregnancy, hepatic insulin signalling is attenuated, which suppresses glycolysis and dysregulates gluconeogenesis, thereby directing more glucose to fetal growth. At the same time, there is an increase in the amount of fatty acid taken up and undergoing β-oxidation by the maternal liver [51,52].

## 2. Gestational Diabetes Mellitus

While insulin resistance is a natural process that occurs during pregnancy, compensation by the endocrine pancreas and peripheral tissues maintain glucose homeostasis until parturition. GDM may develop when compensatory mechanisms are insufficient to overcome the insulin resistance of pregnancy, which is associated with excessive gestational weight gain or obesity that precedes pregnancy [53].

GDM is defined as the new onset of hyperglycemia and glucose intolerance mid-gestation. Worldwide, as many as one in seven pregnancies is affected by GDM [54]. Maternal obesity and advanced maternal age are risk factors for development of GDM [55,56]. As an increasing number of women are beginning pregnancies later in life and there are increasing numbers of obese or overweight women, there has been a corresponding rise in the incidence of GDM. The implications of GDM on maternal health include difficulties with pregnancy (such as pre-eclampsia), complications in labour and delivery and increased risk of postnatal maternal health complications [54,57,58]. Women who have had GDM are at higher risk of developing GDM during subsequent pregnancies, as well as of developing type 2 diabetes (T2D) later in life [59]. Diagnostic criteria can vary between health regions. Undiagnosed GDM due to less stringent diagnostic criteria was associated with higher rates of complications such as excessive gestational weight gain, caesarean delivery, macrosomia and large-for-gestational-age (LGA) infants [60,61]. The only outcome-based guidelines are the International Association of the Diabetes and Pregnancy Study Groups (IADPSG) criteria, which bases diagnosis on a fasting plasma glucose level exceeding 5.1 mmol/L or a plasma glucose level of 8.5 mmol/L 2 h after a 75 g oral glucose tolerance test [62]. Untreated GDM is associated with worse perinatal outcomes [63], and results from the Hyperglycemia and Adverse Pregnancy Outcomes study show more severe insulin resistance and glucose intolerance in offspring of mothers with untreated GDM [64]. Because the increased incidence of GDM reflects the metabolic health burden on women, clinical prevention strategies should target early preventive strategies for risk factors such as maternal obesity. The National Institute for Health and Care Excellence (NICE) prevention guidelines for pregnant women emphasize the importance of planning pregnancy and sharing information about diabetes outcomes and risks for mother and baby with women who are planning to conceive. In addition, the NICE guidelines recommend using the 75 g 2 h oral glucose tolerance test in all women with GDM risk factors in the first and second trimesters, offering continuous glucose monitoring to initiate earlier interventions [65].

During the COVID-19 pandemic, stakeholders and healthcare professionals were concerned about the increased exposure of pregnant women to infection. This resulted in a change in protocol for GDM screening in multiple countries, including in Canada, from a glucose challenge test to an A1C measurement and random, non-fasting blood glucose [66]. It was predicted that this strategy, despite limiting exposure of pregnant women to COVID-19, could potentially lead to missed GDM diagnosis, and a 2022 review of outcomes in Ireland, where similar changes were made, confirmed a significant decrease in GDM diagnoses compared to 2019 [67]. A 2020 review suggested that as many as 80% of GDM cases went undiagnosed in 2020 due to changes in diagnostic strategies during the COVID-19 pandemic [68]. Interestingly, cases of GDM overlooked due to alterations in screening strategies did not lead to worse maternal or fetal outcomes [67,68]. However, women diagnosed in 2020 were more likely to require insulin or metformin at the time of diagnosis and at term than those diagnosed as a result of screening prior to the pandemic [67]. The results of these changes to screening protocols suggest that glucose challenge and glucose tolerance tests are necessary to identify GDM in pregnancy.

The developmental origins of disease theory links in utero environmental exposures to chronic disease and adverse health outcomes later in the life of the offspring [69]. Given this critical stage of fetal development, it is not surprising that GDM also influences the health of the offspring. Data from epidemiological, birth cohort and animal model studies shows that diabetes during pregnancy is associated with the development of cardiometabolic diseases such as obesity, diabetes, high blood pressure, and renal and heart disease later in life [70,71,72,73,74,75,76,77]. For example, the HAPO follow-up study showed a linear relationship between higher maternal blood glucose and impaired glucose tolerance in 10–14-year-old offspring, independent of maternal and childhood BMI and family history of diabetes [78]. Glucose intolerance is also associated with leptin levels in five-year-old children [79]. These findings emphasize the necessity of earlier and more aggressive screening and treatment of GDM.

### 2.1. Impact of GDM on the Liver

Hepatic insulin resistance can contribute to more severe hyperglycemia during pregnancy, with dysregulation of hepatic gluconeogenesis resulting in excessive endogenous glucose production [6]. In addition, hyperlipidemia associated with maternal obesity and GDM can increase hepatic lipid synthesis and overload oxidative capacity, leading to secretion of very low-density lipoproteins (VLDL) with higher triacylglycerol content, as well as ectopic fat deposition in the liver [80]. The resultant lipotoxic and oxidative stress can exacerbate hepatic insulin resistance [81]. There are interesting links between the excessive storage of lipids in the liver, hepatic steatosis and GDM. The prevalence of non-alcoholic fatty liver disease (NAFLD) among women of childbearing age is estimated to be 10% [82]. Women with a history GDM are at higher risk of developing hepatic steatosis later in life [83]. Because hepatic steatosis contributes to insulin resistance, it is significant that development of fatty liver early in gestation (first trimester) can predict glucose intolerance mid-pregnancy [84] and precedes hyperglycemia in GDM [85]. Prospective cohort studies have shown that the presence of elevated visceral adipose tissue, together with sonographically detectable hepatic fat predicted GDM, independent of maternal age, ethnicity, and BMI [84,85]. The inclusion of NAFLD parameters such as hepatic steatosis and liver enzyme levels in an early prediction model for GDM improved the prediction of GDM development in women [86]. While GDM has been identified as a risk factor for the development of NAFLD [87], the mechanisms linking NAFLD to the development of GDM remain to be determined.

### 2.2. β-Cell Dysfunction in GDM 

During pregnancy, β-cells must implement structural and functional changes to overcome insulin resistance and maintain normoglycemia. However, in a subset of women, functional and structural adaptations are impaired, contributing to GDM development. While the specific causes of impaired β-cell compensation in GDM continue to be investigated, this adaptive failure is likely multifactorial, involving genetic variants, nutrient overload and metabolic stress or increased inflammation [15,88,89]. Pancreatic β-cells have been shown to be affected by systemic inflammation [13]. Obese individuals and women with GDM have been found to have higher levels of circulating TNF-α, a proinflammatory cytokine linked to disrupted β-cell function and dedifferentiation [90,91]. Other markers of inflammation such as interleukin 1β (IL-1β) and interferon-γ (IFNγ) are reportedly increased under conditions of metabolic stress [92,93] and can trigger endoplasmic reticulum (ER) stress in β-cells, which prevents adaptation to insulin resistance during pregnancy, contributing to β-cell dysfunction [94].

Pancreatic β-cells are also susceptible to the effects of obesity and nutrient overload. Lipotoxicity and glucotoxicity are potential mechanisms involved in β-cell dysfunction in T2D and GDM [95]. Prolonged exposure to hyperglycemia can lead to impairments in β-cell function and reduced insulin secretion, which can become irreversible [96]. Exposure to high levels of lipids results in lipotoxic β-cell dysfunction [97]. ER stress [98] and oxidative stress [95] are triggered when lipids build up within the pancreatic islet and impair insulin production. Increased oxidative and ER stress can contribute to cell damage and β-cell apoptosis [95].

### 2.3. Impact of GDM on Adipose Tissue

In GDM, insulin resistance in WAT can result in excessive lipolysis, low-grade inflammation and dysregulated adipokine signalling [99]. While a degree of insulin resistance is a physiological adaptation in late gestation, in GDM, significant impairments can occur in downstream insulin signalling in WAT (e.g., decreased insulin receptor-β (IR-β) phosphorylation, downregulation of IRS1 and signalling through PI3K) [100]. Glucose uptake by adipose tissue is also blunted, with defects in GLUT4 translocation observed in GDM [51].

Insulin resistance in adipose tissue also impacts lipid metabolism, and impaired post-receptor signalling resulted in dramatically elevated WAT lipolysis in women with GDM [99]. Adipose tissue expansion is important to accommodate increased lipogenesis in early pregnancy. Without adequate WAT expansion, more severe insulin resistance and ectopic fat deposition in peripheral tissues, as well as adipose tissue inflammation occur [45]. Impairments in WAT expansion has been implicated in GDM [45]. In GDM, adipocytes become larger but show limited vascularization and reduced expression of markers associated with expandability. A proteomic study comparing adipose tissue isolated from women with GDM identified increases in fibrinogen and LUM, the former of which is associated with inflammation and the latter of which is an extracellular matrix protein, indicating adipose tissue rigidity and reduced flexibility [101]. Obesity in pregnancy is associated with increased insulin resistance, dyslipidemia, and inflammation [102]. This leads to a higher risk of endothelial dysfunction and lipotoxicity, worsening insulin resistance and metabolic dysfunction [103,104].

## 3. Adiponectin

Adiponectin is the 30 kD product of the *AdipoQ* gene. The adiponectin protein has an N-terminal signal domain, a variable region, a collagenous domain and a globular C-terminal domain [105,106]. Expression of *AdipoQ* is transcriptionally and epigenetically regulated. Peroxisome proliferator-activated receptor (PPAR)-γ upregulates the expression of *AdipoQ* [107], whereas proinflammatory signals such as TNF-α downregulate its expression [108], and hypermethylation of *AdipoQ* has been associated with altered gene expression and glucose tolerance [109]. Adipose tissue is the major source of adiponectin, and in the obese condition, less adiponectin is secreted into the circulation and levels are lower in dysglycemic individuals [110]. Adiponectin exists in the circulation either in a shorter globular form (gAPN), a full-length form (fAPN) or in multimeric forms of full-length adiponectin, i.e., a low-molecular-weight (LMW) trimer, a medium-molecular-weight (MMW) hexamer or a high-molecular-weight (HMW) oligomer [111]. Multimerization and secretion of adiponectin is regulated by the molecular chaperones ERp44, Ero1-Lα and DsbA-L in the endoplasmic reticulum [112]. There are two adiponectin receptors, AdipoR1 and AdipoR2. AdipoR1 is expressed in skeletal muscle and preferentially binds gAPN over fAPN; AdipoR2 is expressed in the liver [113] and the placental trophoblast and binds both gAPN and fAPN. Notably, the circulating forms of adiponectin and their relative abundance have differing metabolic activities, and the ratios of HMW to total adiponectin may be a biomarker for metabolic dysfunction; lower levels of HMW adiponectin may relate to higher disease risk [111]. Evidence indicates that adiponectin sensitizes tissues to the actions of insulin [105,114,115], and several single-nucleotide polymorphisms (SNPs) in the adiponectin gene in the human population have been associated with higher incidence of diabetes, including the rs2241766 and rs266729 SNPs which are associated with GDM [116].

### 3.1. Adiponectin and Adipose Tissue Function in Pregnancy

Levels of adiponectin decrease progressively throughout pregnancy in parallel with increasing insulin resistance and gestational weight gain [117,118]. In healthy individuals, adiponectin is abundant in early pregnancy, and despite gestational weight gain and the steady decline in insulin sensitivity, decreases in adiponectin levels are modest [102,119], and adiponectin maintains physiological activity (Table 1). Conversely, low levels of adiponectin in all stages of gestation are associated with a higher risk of metabolic dysfunction in pregnancy, increased incidence of GDM and increased risk of adverse outcomes for mother and baby [120,121,122,123]. In fact, low levels of adiponectin early in pregnancy [120,122] or prior to pregnancy [121] are predictive of GDM development. Retnakaran et al., showed that adiponectin may be more closely associated with GDM development and insulin resistance than adiposity or gestational weight gain [124]. Adipose tissue from women with GDM was found to have lower levels of adiponectin, even when adjusted for BMI [109]. Previously, hypermethylation of the human adiponectin gene (*ADIPOQ*) was reported to be associated with obesity [125,126]. Interestingly, Ott et al., also identified hypermethylated regions of *ADIPOQ* in WAT from GDM mothers [109]. Ott et al., also noted significant alterations in adipose tissue *ADIPOQ* methylation in offspring exposed to GDM, suggesting both tissue-specific and heritable epigenetic regulation of adiponectin expression by GDM exposure, even after correcting for maternal BMI and neonatal sex [109].

Experiments performed in 3T3L-1 adipocytes showed a potential mechanism whereby ubiquitination of adiponectin in obesity decreases adiponectin in pregnancy [127]. Ubiquitination and degradation of adiponectin appeared to be prevented by insulin and promoted by inflammation and ER stress, both of which are present in adipose tissue from obese individuals [127]. Adiponectin secretion and mRNA levels decrease in WAT as pregnancy progresses, in parallel with increasing insulin resistance [102]. Insulin positively regulates adiponectin synthesis and release in vitro, and inflammatory cytokines such as TNF-α (which is increased in circulation during gestation) opposes the stimulatory effects of insulin on adiponectin secretion [128]. In adipose tissue, adiponectin has been reported to regulate ‘healthy’ adipocyte expansion and promote an anti-inflammatory phenotype [129,130,131] (Table 1). In pregnant adiponectin knockout mice, WAT has more of the hypertrophic phenotype in the third trimester [132], which has been associated with increased insulin resistance and inflammation [133]. In clinical studies, as well as animal models of GDM, adiponectin is reduced in GDM, when inflammatory markers such as TNF-α and interleukin (IL)-6 are correspondingly increased [134,135,136]. Given the role of adiponectin in the maintenance of healthy adipose tissue, low adiponectinemia in pregnancy may contribute to inflammatory and insulin resistant adipose tissue phenotypes in GDM. 

Lipolysis increases in the later stages of a healthy pregnancy. Adipose tissue becomes increasingly insulin-resistant, leading to reduced insulin-mediated suppression of lipolysis and increased turnover of free fatty acids [137]. A transgenic *ob/ob* mouse model overexpressing adiponectin has been shown to have improved glucose tolerance, reduced serum lipids and hepatic steatosis, possibly due, in part, to the ability of adipose tissue to expand and prevent lipid spillover [138]. Often considered an insulin sensitizer, rodent and cell culture models suggest that adiponectin suppresses lipolysis in adipose tissue and cultured adipocytes [139]. Adiponectin knockout mice show elevated lipolysis [139] and a more hypertrophic adipose tissue phenotype in pregnancy [132]. Still, most studies reporting on the effect of adiponectin on white adipose tissue rely on observations from knockout models [132,139] or adiponectin administration. Although there is abundant evidence for paracrine action of adiponectin in adipose tissue [131,140,141], there is a relative dearth of mechanistically focused studies in comparison to those on endocrine actions of adiponectin.

### 3.2. Adiponectin and Liver Metabolism in Pregnancy

Adiponectin potentiates some effects of insulin in the liver, but does not necessarily “mimic” them. While adiponectin augments the suppression of gluconeogenesis by insulin [142,143], it opposes the effects of insulin on lipid metabolism. Insulin promotes lipogenesis, and adiponectin suppresses lipogenesis and stimulates fatty acid oxidation [144,145]. Acting via the ADIPOR1 receptor, adiponectin requires intracellular signalling through AMP-activated protein kinase (AMPK) activation to inhibit transcription of lipogenic genes by sterol response element-binding protein (SREBP)-1c [144]. Activation of AMPK by adiponectin also inhibits acetyl-CoA carboxylase (ACC), which maintains carnitine palmitoyl transferase-1 (CPT-1) activity, allowing for uptake and oxidation of fatty acids by the mitochondria [106]. Adiponectin reduces endogenous glucose production through the inhibition of phosphoenolpyruvate carboxykinase and glucose-6-phosphatase [142,146,147]. This effect, in combination with reductions in intracellular lipid content, improves hepatic insulin sensitivity and solidifies the role of adiponectin as an insulin sensitizer.

In human studies, low levels of circulating adiponectin have been associated with NAFLD [148,149], and rodent studies show that adiponectin knockout mice develop more severe high-fat-diet-induced hepatic steatosis [150]. Increasing circulating adiponectin levels in rodent models of obesity attenuates hepatic steatosis [151]. Clinical data suggest that reduced adiponectin levels in the first trimester of pregnancy are an independent predictor of the development of GDM [152]. Notably, maternal plasma adiponectin was correlated with both the severity of fatty liver disease and the risk of developing GDM [85]. Pregnant adiponectin knockout mice exhibited increased hepatic triglyceride secretion, as well as increased gene expression of *Dgat1* and *Mttp*, which are key steps in triglyceride synthesis and secretion [153]. In a separate study, it was observed that adiponectin knockout mice developed histologically detectable hepatic steatosis and triacylglycerol accumulation in late pregnancy, even when fed a low-fat diet [132]. This was associated with elevated rates of triacylglycerol synthesis and reduced oxidation of fats in hepatocytes isolated from pregnant adiponectin knockout mice [132]. Hepatic gene expression studies in the livers of pregnant adiponectin knockout mice showed increased levels of the regulatory genes of hepatic gluconeogenesis, *Pck1* and *G6pc* [132,153]. Correspondingly, hepatic *de novo* lipogenesis was increased, and hepatic glucose output was dysregulated, which did not respond adequately to insulin administration [132,153]. Increasing circulating adiponectin in mid-gestation reduced hepatic lipid accumulation and improved glucose tolerance in both low-fat- and high-fat-fed adiponectin knockout mice [132]. These findings suggest that adiponectin has a significant role in regulating glucose homeostasis during pregnancy by regulating hepatic lipid metabolism and gluconeogenesis.

### 3.3. Regulation of Endocrine Pancreas Hormone Secretion by Adiponectin during Pregnancy

**Figure 2:**
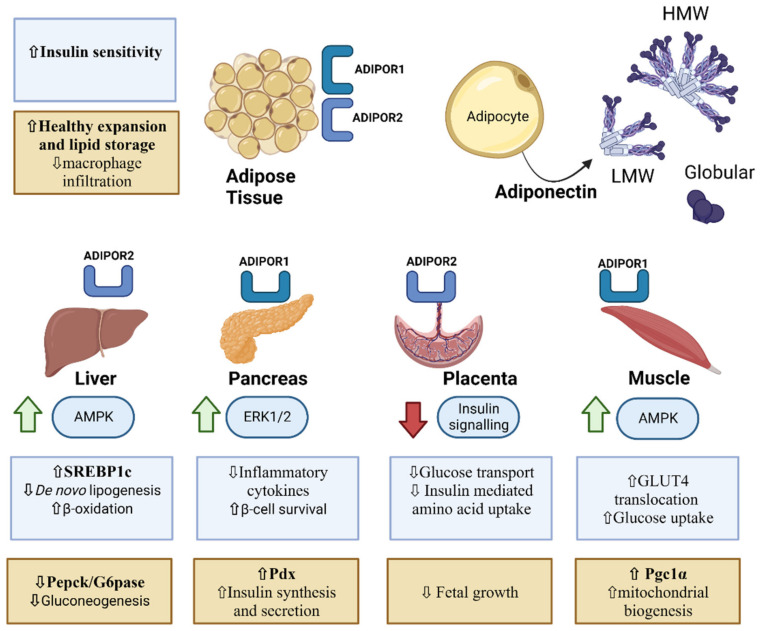
Impact of adiponectin signalling on metabolism and fetal growth. Adiponectin is secreted from adipocytes and exists in circulation in a variety of forms, including low-molecular-weight (LMW) trimers bound by disulphide bonds; large, high-molecular-weight (HMW) complexes; and globular adiponectin that has been proteolytically cleaved from the collagen domain. These isoforms have different tissue and receptor specificity and are postulated to have varying metabolic activity. Adiponectin receptors ADIPOR1 and ADIPOR2 are present in differing levels of abundance in insulin-sensitive tissues [112]. White adipose tissue contains both ADIPOR1 and ADIPOR2 [162], and increasing adiponectin signalling in WAT has been shown to increase insulin sensitivity, improve healthy expansion and decrease inflammation and macrophage infiltration. Adiponectin signalling in the liver occurs through ADIPOR2 and in the pancreas through ADIPOR1, and both ADIPOR1 and ADIPOR2 have been identified in the placenta [163,164,165]. Adiponectin increases AMPK signalling in the liver and skeletal muscle, leading to increased glucose uptake and increased mitochondrial biogenesis in skeletal muscle, as well as improved lipid metabolism and decreased glucose output in the liver [144]. In the pancreas, increased signalling through ERK1/2 downstream of adiponectin leads to decreased inflammation, resulting in improved β-cell survival and insulin synthesis and secretion [154,155,157]. In contrast, placental adiponectin signalling from the maternal side promotes reduced glucose transport and decreased insulin signalling and insulin-mediated amino acid transport, which is postulated to contribute to reduced fetal growth [111]. ↑ increased, ↓ decreased. Created with BioRender.com.

Adiponectin receptors have been identified in β-cells, suggesting functional adiponectin signalling [154,155,156]. Adiponectin signalling also has a role in the maintenance of β-cell mass and, potentially, function (Figure 2) [153,157,158,159,160]. Low circulating adiponectin levels are associated with impaired β-cell function in pregnant women [159]. Qiao et al., observed impairments in maternal β-cell adaptations to pregnancy, including marked smaller islets and lower β-cell mass in pregnant adiponectin knockout mice relative to wild-type animals [153]. Supplementation with adiponectin in mice can increase β-cell mass and circulating insulin during pregnancy [153]. During pregnancy, adiponectin was also reported to promote β-cell expansion via increased placental lactogen expression [161]. These data implicate a role of adiponectin in structural β-cell adaptations to pregnancy.

Whether or not adiponectin acts to improve glucose-stimulated insulin secretion is contested in the literature [160,166]. Okamoto and colleagues [166] showed increases in adiponectin-stimulated insulin secretion by isolated mouse islets under low-glucose conditions. Following glucose load, impaired insulin secretion was observed in non-pregnant adiponectin KO mice fed a standard diet [167]. Therefore, adiponectin may be involved in insulin secretion in pregnancy [153], and adiponectin deficiency may lead to impairments in the ability of the islet to adapt to metabolic stress in pregnancy. In a drug-induced polycystic ovary syndrome model, islet expansion and increased insulin gene expression in response to insulin resistance were observed in adiponectin knockout mice, suggesting that in the absence of a second metabolic hit such as diet or pregnancy, the pancreatic islet of adiponectin knockout mice produces adequate insulin [168]. The precise role of adiponectin in the adaptation of the islet to pregnancy or whether there is any impact of adiponectin on glucagon secretion has yet to be determined. These studies suggest that adiponectin plays a key role in the adaptive metabolic response to pregnancy.

**Table 1:** Physiological actions of adiponectin during pregnancy.

Effect of Adiponectin	Tissue	Reference
Healthy pregnancy		
Healthy expansion of adipose tissue, adipogenesis, ↑ lipid storage, inhibits lipolysis, ↑ glucose uptake in 3T3-L1, improves insulin sensitivity	Adipose Tissue	[106,111,131,153]
↓ Lipogenesis ↓ gluconeogenesis ↑ beta oxidation	Liver	[106,132,153]
↓ Apoptosis ↑ insulin secretion ↑ beta-cell mass	Endocrine Pancreas	[39,111,153,157,158,161]

↓ decreased, ↑ increased.

### 3.4. Placental Adiponectin

There are conflicting reports about whether the placenta expresses and secretes adiponectin [163,169]. However, more sensitive techniques have determined that the placenta does not produce endogenous adiponectin [119,170,171]. Adiponectin in the maternal circulation does not cross the placenta; however, adiponectin receptors (ADIPOR1 and ADIPOR2) are present in the placenta [163,165,172]. Placental signalling by adiponectin receptors impacts nutrient transport and fetal growth [34,173]. Adiponectin is likely compartmentalized in the maternal and fetal systems and signals through the placenta via adiponectin receptors on the trophoblast [34,163,165,172,174] (Figure 2). Adiponectin signalling in maternal tissues appears to differ greatly from signalling in the placenta. In vitro studies show that both full-length and globular adiponectin may suppress placental insulin signalling by reducing phosphorylation of IRS-1, AMPK and AKT through activation of PPAR-α [175]. A consequence of reducing insulin sensitivity at the level of the placenta is a reduction in amino acid transport, which has implications for fetal growth [34,175]. A recent study using adiponectin knockout and heterozygous mice revealed that reduced adiponectin impacted placental nutrient transport, primarily fatty acids [176]. Treating placental trophoblasts with adiponectin in vitro, or chronic infusion of adiponectin to pregnant mice attenuates placental insulin signalling and reduces fetal growth [34]. These studies outline a direct role of adiponectin signalling and placental nutrient uptake.

### 3.5. Adiponectin in the Fetus

While maternal adiponectin decreases throughout gestation, fetal adiponectin increases, promoting fat deposition and fetal growth [177]. Fetal adiponectin is postulated to have the opposite effect of circulating adiponectin in adults. For example, an association between increased cord blood adiponectin and increased childhood adiposity at 3 years of age was found in the project VIVA cohort [178]. Fetal adiponectin appears to promote fetal growth and may potentiate insulin resistance [36,179]. Consistent with this, fetal adiponectin and maternal blood glucose levels are inversely correlated in the second trimester [12,37]. In rodent studies, fetal adiponectin was found to promote adiposity [177]. Administration of full-length adiponectin increased litter size and decreased neonatal size in mice [173]. This suggests that maternal adiponectin regulates fetal growth, potentially by downregulating placental amino acid transport and insulin/mTOR signalling [173,180]. In contrast to maternal adiponectin, fetal adiponectin serves to promote fetal growth and adipose deposition, possibly by increasing fetal insulin resistance [36]. Fetal fat deposition was impaired in fetuses from adiponectin-null dams but not in heterozygous mice [177]. There is also an inverse relationship between maternal adiponectin and fetal growth, with low circulating adiponectin associated with LGA infants [181]. Maternal administration of adiponectin reduces fetal growth by impacting placental amino acid transport [173] and, potentially, by increasing IGFBP-1, which was reduced in adiponectin-deficient offspring and is inversely correlated with birthweight. As the effect of adiponectin on fetal growth is proposed to be a consequence of altered placental nutrient transport because adiponectin does not cross the placenta [111], the impact of exogenous adiponectin on fetal growth is likely limited by nutrient availability or, as in GDM, by oversupply of nutrients.

In rodents, fetal adiponectin also impacts lipid metabolism. In contrast to maternal adiponectin, fetal adiponectin increases circulating free fatty acids and hepatic lipogenic genes [177]. While maternal obesity increases adiposity in the offspring, the expression of adiponectin is also increased in the adipose tissues of the offspring [182], suggesting that the regulation of adiponectin levels is altered by the maternal environment. This concept is supported by the observation that at birth, cord blood adiponectin is as much as seven times higher than maternal levels but decreases significantly within the first year of life [183]. Mouse studies using diet-induced obesity show that administration of adiponectin reduces fetal weight relative to sham infusion, indicating a role of adiponectin in fetal growth [111]. A more recent study followed offspring to nine months of age and determined that maternal supplementation with adiponectin in late gestation improves glucose tolerance and insulin sensitivity in offspring born to obese dams [184]. 

Experiments using adiponectin knockout models have demonstrated that the lack of adiponectin in pregnancy has varied impacts on fetal growth and development. Qiao et al., reported that chow-fed adiponectin-null dams had heavier fetuses [31,153]. However, the same group previously reported that adiponectin deficiency increased fetal weight [153,185] and reduced fetal weight relative to heterozygous controls [177]. Shreshta et al., did not observe any effect of adiponectin deficiency (heterozygous reduction or complete knockout) on fetal weight [176].

### 3.6. Treatment of Metabolic Disease during Pregnancy with Adiponectin

The action of adiponectin in pregnancy has been solidified by proof-of-concept supplementation studies in animal models, including the use viral vectors and agonists that increase downstream signalling pathways implicated in adiponectin action [185]. There is no published evidence for supplementation in humans or whether direct supplementation with adiponectin is a feasible therapeutic option for GDM.

Experiments in rodent models have shown the metabolic benefit of increasing circulating adiponectin in pregnancy. Adenoviral-mediated adiponectin delivery mid-gestation ameliorated the development of hepatic steatosis and attenuated hyperglycemia in pregnant adiponectin-null mice [132]. Moreover, Qiao et al., showed that increasing adiponectin during pregnancy in adiponectin-null mice restored β-cell mass and increased circulating levels of insulin [153]. Using adiponectin knockout mice, Aye et al., determined that sustained supplementation with adiponectin during pregnancy could prevent the metabolic programming that leads to placental dysfunction and altered fetal growth in obese dams [111]. Although these results are promising, supplementation in rodent models often utilizes perfusion systems [111] or hydrodynamic [186] or viral gene delivery systems [132,153] to maintain circulating adiponectin levels due to rapid turnover of adiponectin in circulation [187]. Additionally, multiple isoforms of adiponectin exist in different ratios, with varying metabolic activity and post-translational modifications of adiponectin can impact optimal bioactivity, which may differentially impact both maternal and offspring metabolic health outcomes [127]. 

To date, there have been no studies evaluating the effect of adiponectin supplementation in humans, although the effect of existing drugs and natural health products on adiponectin levels is an active area of research [188]. In pregnancies affected by GDM, diet and lifestyle interventions are often prescribed to control hyperglycemia without the use of medications [189]. Weight loss and exercise [190,191,192] also increase adiponectin levels. Thus, increased adiponectin could be a mechanism underpinning exercise- and weight-loss-induced improvements in metabolic health in women with GDM. Pharmacological therapies for diabetes, including rosiglitazone and pioglitazone, from the thiazolidinedione (TZD) class of PPAR-γ agonists, also increase circulating adiponectin [193]. Indeed, increased circulating adiponectin is thought to mediate the action of pioglitazone [167], and adiponectin-null mice exhibit reduced responsiveness to PPAR-γ agonists [194]. However, TZDs are not used in pregnancy and are categorized as class C for teratogenicity and, as such, are not a viable treatment option for GDM [195]. 

In contrast, metformin is commonly used prior to pregnancy and throughout gestation, along with insulin, as a first-line therapy for GDM [196]. Both in vivo and in vitro results using adipose tissue explants from diabetic and non-diabetic patients showed that the drug metformin was capable of increasing adiponectin gene expression [197]. Incretin-based therapies for diabetes such as dipeptidyl peptidase (DPP)-4 inhibitors or GLP-1 receptor agonists have also been experimentally shown to increase adiponectin synthesis and release both in vitro and in patients with T2D [198]. Like TZDs, DPP-4 inhibitors are contraindicated in pregnancy [199]; however, a recent systematic review showed that DPP-4 inhibitors and GLP-1 receptor agonists have the potential to reduce the risk of postpartum T2D and improve β-cell function in patients with GDM [200]. Adverse outcomes have been reported in animal models of pregnancy with the use of GLP-1 receptor agonists [201]. However, evidence implicates adiponectin in β-cell adaptation and survival in pregnancy, as well as glucose homeostasis, so other strategies to increase circulating adiponectin or downstream adiponectin signalling safely in pregnancy could be effective therapies for GDM. 

The use of adiponectin as a therapeutic has been largely ruled out due to challenges with protein heterogeneity, solubility, and post-translational modifications [202]. AdipoRon is a small-molecule adiponectin receptor agonist that has been shown to improve glucose homeostasis, insulin resistance and dyslipidemia in *db/db* mice when administered orally [203]. AdipoRon also protected against lipotoxicity in the hearts of *db/db* mice [204]. In 2021, a study in pregnant rats showed that AdipoRon reduced hyperglycemia in dams with GDM and improved long-term glucose tolerance in the offspring of pregnant streptozotocin-induced diabetes treated with AdipoRon [205]. While experiments using AdipoRon in pregnant diabetic rats by Gazquez et al., are an excellent starting point, this model involves β-cell destruction to induce hyperglycemia, which represents a type 1 diabetes model of diabetes in pregnancy [206]. It would be informative to determine whether AdipoRon can ameliorate the effects of high-fat or high-fat and sucrose diet feeding and maternal obesity in pregnant animal models. Future research on AdipoRon should include additional studies on human cells and tissue, as well as studies in animal models, to determine whether AdipoRon could prevent the long-term damaging effects of GDM exposure on offspring.

## 4. Summary and Future Perspectives

GDM is one of the most common complications of pregnancy. Its increasing global prevalence and the association of GDM with perinatal complications and long-term metabolic and cardiovascular disease risks is a major cause for concern. While there would be benefit in preventing GDM, early pregnancy-predictive criteria are not yet practical. Therefore, new therapeutic options for women with GDM are needed.

Current research has established the role of adiponectin in whole-body energy homeostasis, including the maintenance of a healthy pregnancy. Studies suggest that adequate levels of adiponectin in pregnancy may counter the effects of insulin resistance, preventing the development of hyperglycemia and GDM. While the mechanisms underlying the insulin-sensitizing properties of adiponectin have been outlined using cellular and animal models, few studies have examined the tissue-specific effects of the adiponectin receptors in pregnancy. For example, while the effects of adiponectin on skeletal muscle metabolism and insulin sensitivity are well-established, its effects on skeletal muscle during pregnancy have not been explored. In addition, the insulin-sensitizing properties of adiponectin supplementation remain to be examined in pregnant women; however, challenges regarding administration and dosages would need to be settled to achieve a successful clinical trial. Adiponectin therapy could also be useful in the immediate postnatal stage to decrease the progression from GDM to T2D in women. In this regard, further studies during pregnancy of molecules that stimulate adiponectin signalling, such as AdipoRon, could be a more successful approach.

Another important area for future research is clarification of the role of placental and fetal adiponectin in metabolic homeostasis, and tissue-restricted and inducible mouse model systems could help in this regard. Moreover, it is unclear whether low circulating adiponectin has a causative role in the GDM-induced developmental origins of obesity and insulin resistance in offspring, although studies involving adiponectin supplementation suggest it is protective [111,132,153,173]. Both the effects on the fetus and the long-term effects of small molecules that activate adiponectin signalling need to be examined before they can be utilized in clinical trials.

## Data Availability

Not applicable.
